# A Comparative Analysis of Burned Area Datasets in Canadian Boreal Forest in 2000

**DOI:** 10.1155/2013/289056

**Published:** 2013-05-30

**Authors:** Laia Núñez-Casillas, José Rafael García Lázaro, José Andrés Moreno-Ruiz, Manuel Arbelo

**Affiliations:** ^1^Grupo de Observación de la Tierra y la Atmósfera (GOTA), Universidad de la Laguna, 38200 San Cristóbal de la Laguna, Spain; ^2^Departamento de Informática, Universidad de Almería, 04120 Almería, Spain

## Abstract

The turn of the new millennium was accompanied by a particularly diverse group of burned area datasets from different sensors in the Canadian boreal forests, brought together in a year of low global fire activity. This paper provides an assessment of spatial and temporal accuracy, by means of a fire-by-fire comparison of the following: two burned area datasets obtained from SPOT-VEGETATION (VGT) imagery, a MODIS Collection 5 burned area dataset, and three different datasets obtained from NOAA-AVHRR. Results showed that burned area data from MODIS provided accurate dates of burn but great omission error, partially caused by calibration problems. One of the VGT-derived datasets (L3JRC) represented the largest number of fire sites in spite of its great overall underestimation, whereas the GBA2000 dataset achieved the best burned area quantification, both showing delayed and very variable fire timing. Spatial accuracy was comparable between the 5 km and the 1 km AVHRR-derived datasets but was remarkably lower in the 8 km dataset leading, us to conclude that at higher spatial resolutions, temporal accuracy was lower. The probable methodological and contextual causes of these differences were analyzed in detail.

## 1. Introduction

Different research centers and institutions have worked on burned area (BA) mapping on regional and global scales. However, there are still technical limitations linked to the remote sensing imagery, classification methods, and classification challenges based on differing environments. The spectral, spatial, and temporal resolutions of the remote sensing data; the trade-off between accuracy and adaptability of BA detection algorithms; and different vegetation dynamics and postfire recovery patterns are some of the factors that may result in varying levels of efficiency of a given methodology. Some periods of time were particularly unfavorable to BA detection for different reasons, affecting the continuity of BA time series datasets and highlighting the strengths and weaknesses of BA detection methods at the same time. For instance, the year 2000 was a year of low global fire activity [1], and fires in Canada did not produce extremely large burn scars to the large boreal forest belt compared to other years. Instead, fires tended to affect several areas of moderate expanse. Furthermore, during the year 2000 NASA's Moderate-Resolution Imaging spectroradiometer (MODIS) and the Advanced Very High Resolution (AVHRR) instruments produced some unreliable or insufficient information. On the other hand, the year 2000 was considered a turning point for coarse to moderate-resolution datasets in BA mapping, with the advent of 500 m MODIS data after the first two years of 1 km imagery from the VEGETATION (VGT) sensor onboard SPOT-4 satellite. This combination of facts makes it interesting to assess the accuracy of the BA data available for this particular year in the North-American boreal forest, whereas a comparative study would help in analyzing different methodologies during a period of transition to finer spatial resolution imagery.

NASA's projects of the Pathfinder 8 km Land dataset (PAL) and the subsequent Land Long Term Data Record (LTDR) were two significant attempts at achieving a consistent coarse-resolution imagery time series on a global scale from 4 km AVHRR data. The PAL time series from 1984 onwards has been used in several works related to BA estimation [2, 3]. The recently released 5 km BA dataset for the Canadian boreal forests from 1984 to 1999 from LTDR data [4] showed a spatial accuracy comparable to the 1 km BA product from AVHRR-Local Area Coverage (LAC) data published by Chuvieco et al. in 2008 [5] for the years 1984–2006. With the latest version of LTDR (which is still not available for the years after 2000) it will be possible to expand the time series until the present day.

Several global initiatives have coordinated the work done in different countries in order to provide world BA maps from other sensors. In 2001, the GLOBSCAR project launched by the European Spatial Agency (ESA) produced monthly 1 km BA maps from the year 2000 onwards by analyzing data from the moderate-resolution sensor Along Track Scanning Radiometer-2 (ATSR-2) [10]. The following year, the Joint Research Centre (JRC) and other research centers from different countries launched the Global Burned Area 2000 (GBA2000) initiative, a BA map of global coverage from SPOT-VGT data [11]. In reality, however, both projects were in fact limited to the year 2000 and remarkable discrepancies were found between them [1]. The GBA2000 dataset progressively evolved into a new product named L3JRC which would provide a wider temporal coverage until 2007 [8]. Soon after, ESA's GLOBCARBON project integrated efforts from different workstations and the acquired knowledge from GLOBSCAR to provide BA datasets from 2000 to 2007, generated from the same ATSR-2, AATSR, and MERIS sensors on the Envisat platform in addition to the VGT instrument [12]. However, the project ended in 2006 and BA data is no longer available. Finally, the MODIS Collection 5 BA product (MCD45A1) was to become the first global BA data provided from satellite imagery at 500 m spatial resolution and an approximate date of burn in Julian days from the year 2000 [13]. Nevertheless, the MODIS sensor is known to have presented calibration problems during its first year, suggesting that an accuracy assessment would be useful as well. 

An actual comparative analysis of spatial and temporal accuracy from the diverse BA products available for the year 2000 in the Canadian boreal forest has not yet been conducted. Pros and cons of each dataset will be strongly related to inherent features of the corresponding remote sensing imagery, as well as to the particular approaches chosen to solve BA detection challenges and to the regional differences of forest ecology and fire dynamics. From these observations the questions of where, when, and how efficiently each dataset detects BA in the boreal forest of Canada are discussed. Firstly, some ecological patterns of the Canadian boreal forest that might influence BA detection are described, as well as the different methodologies that have been used to detect BA in each analyzed dataset. Secondly, a spatial accuracy assessment in a global, fire-by-fire, and regional approach is presented. Finally, the burn date estimates are considered by means of a spatiotemporal analysis and an overall accuracy assessment. The spatiotemporal accuracy patterns found in each dataset, as well as their probable causes, were summarized in five sections: the relationship between fire size and imagery spatial resolution, the different combinations of remote sensing data depending on each BA detection method, use of fixed and dynamic radiometric thresholds, geographic patterns that might be linked to ecological factors; and considerations regarding the temporal shifts and variability.

## 2. Canadian Boreal Forest

Coniferous species are dominant in the North-American Boreal forests and they vary with latitude and from east to west. Moisture and temperature are general constraints that determine the stand density and species composition. In the highest latitudes, there is a transition zone between the boreal forest and tundra biome. Predominant tree species are black spruce (*Picea mariana *(Mill.) BSP), paper birch (*Betula papyrifera *Marsh.), trembling aspen (*Populus tremuloides *Michx.), balsam fir (*Abies balsamea *(L.)), jack pine (*Pinus banksiana *Lamb.), white spruce (*Picea glauca *(Moench) Voss.), and tamarack (*Larix laricina *(Du Roi) K. Koch). Black spruce, white birch, and aspen are found across the entire boreal forest. Northward, black spruce forms open forests [14], balsam fir is present to the east, lodgepole pine (*Pinus contorta *Dougl. ex. Loud.) is widely distributed in the west, and jack pine is found on both sides [15]. Fire plays an important role in the landscape and has different effects depending on the region by favoring the development of different species after disturbance [16]. Highly energetic crown fires and species with regeneration capacity are the most frequent combination [17]. However, surface fires of medium intensity may occur where trees are sparse or crowns are high above the ground [18]. 

## 3. Burned Area Data

Active fire detection is based on changes in the thermal bands, whereas BA mapping usually depends on the availability of radiometric data in the green (0.5-0.6 *μ*m) and red (0.6-0.7 *μ*m) bands plus the NIR (0.75–0.9 *μ*m) and/or SWIR (1.5–2.0 *μ*m) bands. BA mapping algorithms take into account the differences in these spectral regions between prefire and postfire situations, either through abrupt changes within a sliding temporal window, between discrete units of time, between dates before and after the end of the fire season, or the same dates in different years. The datasets assessed were as follows: the Riaño et al. [2] algorithm applied to PAL data; the Moreno-Ruiz et al. [4] algorithm applied to LTDR v.3 data, the Chuvieco et al. [5] BA dataset from LAC data, the GBA2000 and L3JRC datasets from SPOT-VGT, and the MCD45A1 dataset from MODIS (Table 1). The first three will hereafter be referred to as PAL.ba, LTDR.ba, and LAC.ba, respectively.

### 3.1. PAL.ba from the Riaño et al. Algorithm (8 km)

Riaño et al. [2] used 7-day composites from the daily PAL dataset [19, 20]. The BA algorithm (BAA) used three different types of threshold in order to detect changes along the time series: a temporal threshold checked a simultaneous decrease of vegetation indices VI3T [21] and GEMI [22] indices and an increase in AVHRR channel 3 brightness temperature; a fixed threshold was adjusted based on the knowledge of the land cover, separability tests, and postfire conditions; and a third type of threshold was automatically set by statistical calculations from a vegetation index distribution. Monthly burn scar maps with a spatial resolution of 8 km were then created.

### 3.2. LTDR.ba from the Moreno-Ruiz et al. Algorithm (5 km)

The Moreno-Ruiz et al. [4] algorithm was applied to 10-day composites from LTDR version 3 daily data [23, http://ltdr.nascom.nasa.gov/ltdr/ltdr.html]. The algorithm combined radiometric thresholds, a Bayesian network classifier [24–26], and a postclassification analysis of neighborhood conditions. The algorithm identified the date of fire as the first step in calculating a set of prefire and postfire statistics from several vegetation indices and spectral bands and then estimated the probability of a pixel to have been burned. The last step accounted for neighborhood conditions in every burned pixel in order to improve spatial coherence and to avoid false detection. A 5 km BA dataset was generated with a temporal resolution of 10 days. 

### 3.3. LAC.ba from the Chuvieco et al. Algorithm (1.1 km)

The Chuvieco et al. [5] algorithm was applied to daily AVHRR-LAC data from Latifovic et al. [27]. Spectral bands and derived indices from 10-day composites [6] were fed into the algorithm, a multivariable thresholding approach adapted to Canadian territory. Different thresholds accounted for considerable prefire activity of vegetation; sharp and persistent changes before/after burn; higher temperatures and lower reflectance (bands 1 and 2) after fire; separability with respect to water, dark soils, and so forth. Postprocessing steps eliminated small polygons and applied more relaxed thresholds to pixels surrounding burned detections. 

### 3.4. GBA2000 (1.1 km)

This regional algorithm used daily SPOT-VGT-S1 data in Canada [28, 29]. Training areas were obtained by applying the Li et al. [18] boreal active fire detection algorithm to daily AVHRR and a postfire season SPOT-VGT image. BA detection was based on a multiple logistic regression analysis that used a set of variables to explain a dichotomous independent variable with a value of 1 (burned) or 0 (unburned). The predictor variables were chosen from 10-day and 30-day changes in SPOT-VGT red, NIR, and SWIR bands; NDVI; and a SWIR-based vegetation index (SWVI) [7]. Each change was previously normalized with respect to similar background vegetation (in order to distinguish changes due to seasonality of phenological cycle) and was differentiated according to ecozones.

### 3.5. L3JRC (1 km)

Modifications from the GBA2000 method were developed by Ershov and colleagues [29, 30]. A daily pixel contamination product was created accounting for snow, clouds, shadows, satellite view angle, and fire smoke. Abrupt change detection was analyzed by iterative comparisons of daily NIR (0.83 *μ*m) reflectance values to an aggregation of historical NIR values, according to a temporal index [8]. Tests were applied to the eligible pixels based on statistics of the temporal index within a 200 × 200 spatial window of valid pixels and on fixed thresholds at 0.83 *μ*m and 1.66 *μ*m. False detections for that year were amended by comparison with the Global Land Cover 2000 database [31]. The resulting product was a 1 km daily BA dataset. 

### 3.6. MCD45A1 (500 m)

Imagery from the year 2000 shows striping in bands 5, 6, and 2, which constitute some of the key data in burned area discrimination (http://modis-fire.umd.edu/BA_methodology.html; http://mcst.gsfc.nasa.gov/index.php?section=15). The BRDF (Bi-directional Reflectance Distribution Function) [9] is the basis of change detection here, whereas *Z*-score calculations account for the persistence of change. Burned cell classification is based on temporal changes in daily surface reflectance of NIR and MIR bands. An iterative process predicts reflectance values for each day based on a number of previous days within a temporal window, and the error-weighted difference between predicted and observed values acts as a sign of change. A temporal window of ±8 days establishes the detection precision of burn date estimates, in order to compensate for the effects of missing data [13].

### 3.7. Canadian Forest Service National Fire Database (CFSNFD)

All of this data was compared to the CFSNFD [32]. Only fires larger than 5,000 ha occurring in the year 2000 were studied. Burn scars in this database are mapped by different Canadian fire management agencies using a variety of means including remote sensing, helicopter GPS, ground-based GPS, aerial photography, and aerial sketching. However, the mapping detail in the CFSNFD should be comparable to the detail that would be expected from coarse to moderate-resolution remote sensing data sources, as performed in this study, and has the advantage of providing burn dates that do not depend exclusively on the availability of satellite data on the date the fire started. CFSNFD polygons typically map the outer fire perimeters and do not remove unburned islands and lakes. In this regard, fires selected from the CFSNFD in this study were checked against postfire Landsat imagery in order to visually confirm that no inner lakes or green islands of significant size were included. We used updated BA information from the CFSNFD point version (last released in August 2010), where initial BA estimates were improved with respect to initial fire perimeter delineation.

## 4. Accuracy Assessment Methods

**Figure 1:**
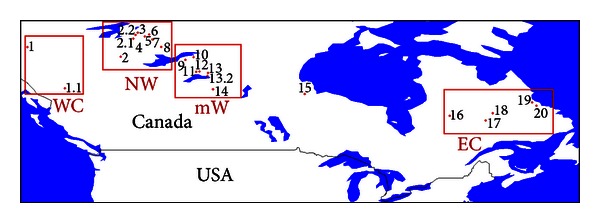
Study area: test sites location and distribution of regional patterns in the entire Canada. The LAC region (upper left corner: 62°16′N, −122°20′E; bottom right corner: 53°N, −96°30′E) contains fires #2 and #8 to #14. Note: fire #11 includes two adjacent fires which were registered separately in the database. Sites were grouped into four geographic regions: West Coast (WC), North-West (NW), mid-West (mW), and East Coast (EC).

Selected BA fires from the CFSNFD were identified in every dataset assessed. The test sites were numbered eastwards from west to east (1 to 20). Sites named as #1.1, #2.1, #2.2, and #13.2 did not appear in the polygon version of the database and were added from the point version. Fire-by-fire comparisons of BA estimates allowed the detection of four regional patterns, which were analyzed as well: West coast, North-West, mid-West, and East coast (Figure 1).

### 4.1. Spatial Analysis

First spatial accuracy assessment was performed on a grid of 40 km squared cells using the Lambert Conformal Conic (LCC) projection, in order to minimize distortions and to keep the shape of the squares equal [5]. BA was calculated for every 40 km cell in the reference map and was contrasted with the same calculations in each of the other BA maps by means of a simple regression model [33]. 

The remaining procedures were implemented in a fire-by-fire analysis approach and based on relative analyses rather than absolute BA detections. The different spatial coverage of the LAC.ba determined the repetition of all the analyses for both the entire Canada and the central spatial subset corresponding to the LAC data frame. First, the relative error was calculated for every fire by comparison against the same fire scene in the reference map, as follows:

(1) RelativeErrori =(ClassifiedBurnedAreai−BurnedAreai)BurnedAreai.

Then, for the relative error distribution, we calculated the weighted means and standard deviations, where each scene's weight was considered as a proportion of the BA of the scene with respect to the sum of all scenes:

(2) μ=∑RelativeErrori∗BurnedAreaiTotalBurnedArea,σ=∑(RelativeErrori−μ)2∗BurnedAreaiTotalBurnedArea.

The error matrix (*P*_*ij*_) [34] and its derived indices (commission and omission errors) were computed at scene level, so as to prevent georeference-derived errors. Considering a dichotomous classification of classes, *C*_1_ (burned) and *C*_2_ (unburned), each element of the error matrix was obtained from the expression

(3) Pij=∑k=1N(pij)kN,

with (*p*_*ij*_)_*k*_ representing the proportion of the BA in the zone *k* assigned to the class *C*_*i*_ which actually belongs to class *C*_*i*_. To compute the error matrix elements we compared the classified percentage of BA (*c*_1_) and the reference percentage (*r*_1_) and assigned the least value as the percentage of the correctly classified (*p*_11_) class [35]. The absolute value of the difference is distributed, either in the percentage of the burned class classified incorrectly (*p*_21_) or in the percentage of the nonburned class classified as burned (*p*_12_), by means of a simple algorithm:

 IF (*c*_1_ ≥ *r*_1_)
 THEN 
 
*p*_11_ = *r*_1_
 
*p*_12_ = *c*_1_ − *r*_1_
 
*p*_21_ = 0
 
*p*_22_ = *c*_2_
 ELSE 
  
*p*_11_ = *c*_1_
 
*p*_12_ = 0
 
*p*_21_ = *r*_1_ − *c*_1_
 
*p*_22_ = *r*_2_
 END_IF.

Thus, the derived indices of interest from the error matrix [36] were computed:

(4) user's  accuracy  of  class  C1=P11P1+,  whereP1+=P11+P12,producer's  accuracy  of  class  C1=P11P+1,  where  P+1=P11+P21,commission  error  of  class  C1  =1−user's  accuracy  of  class  C1,omission  error  of  class  C1  =1−producer's  accuracy  of  class  C1.

### 4.2. Timing Analysis

The temporal distribution of estimated BA of each dataset was represented in a timing histogram obtained from all of the burned cells in each dataset. As temporal resolution varies between different datasets, both monthly and 10-day-composite distributions were built getting the best possible level of detail in each case for the temporal analysis. Burn dates were missing in some of the eastern sites in the reference data, so the 5th percentile from all other products was used for the time distribution. For the largest sites we assumed that the entire area could not have burned on the starting date of the fire, so a proportional amount of the BA estimation was shifted to the following time period. 

## 5. Results 

### 5.1. Spatial Accuracy Assessment

**Figure 2:**
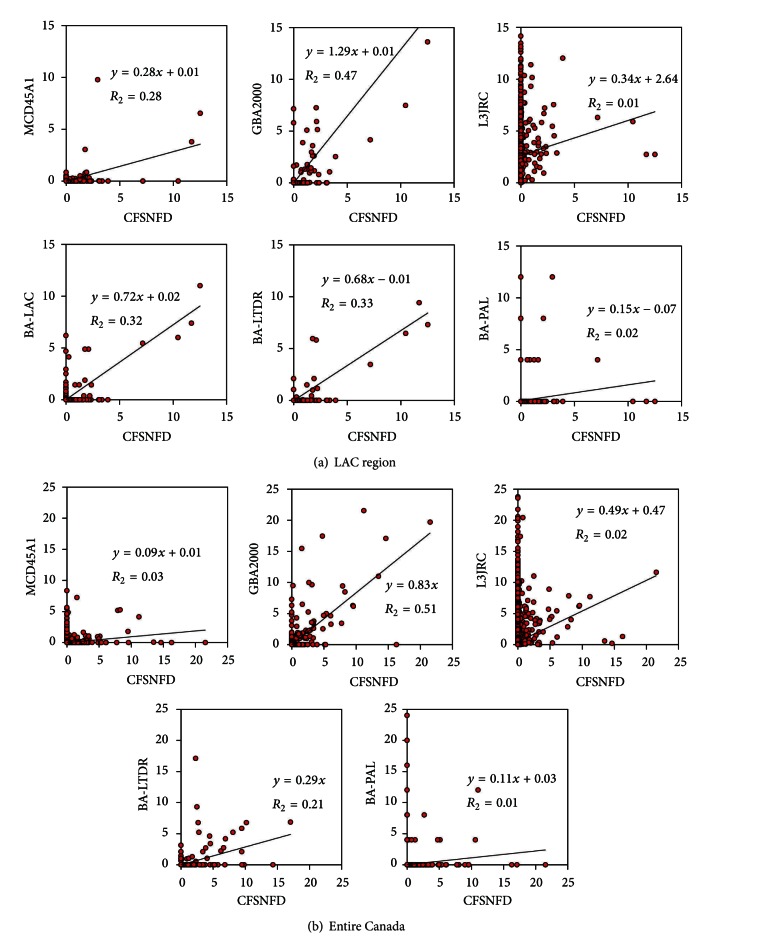
Linear regression of BA proportion (%) in 40 km^2^ from MCD45A1, GBA2000, BA-LAC, BA-LTDR and BA-PAL against BA proportion (%) in CFSNFD for the year 2000: (a) in the LAC region; (b) in Canada.

In this study the area burned in the boreal forests of Canada was underestimated by all the remote sensing products when compared to the CFSNFD for the same year, 2000. The coefficient of determination from the regression model was under 0.6 in all cases, as shown in the scatter plots depicted in Figure 2. The best spatial correlation was found in GBA2000; LAC.ba and LTDR.ba showed very similar behavior in the common areas; and the remaining datasets presented lower spatial correlation, which normally improved in the central spatial subset with respect to the whole territory, except for GBA2000 by overestimation and L3JRC by underestimation.

Relative error distributions of BA estimations in the fire-by-fire analysis are specified in Table 2, by their corresponding weighted mean (*μ*) and standard deviation (*σ*) values. The smallest *μ* was found in GBA2000 (−16%, the negative sign meaning underrating) and the greatest was found in PAL.ba and MCD45A1 (<−75%), the two extremes in spatial resolution. In general, the lower the *μ*, the higher the *σ*, which in GBA2000 was remarkably higher than the others (around 40%). 

Table 3 shows BA estimations for every fire, and the regional patterns are shown in Table 4. In general, fires below 8,000 ha were hardly detected by any AVHRR-derived datasets. However, the latter together with GBA2000 usually gave the best estimates of large burned areas. On the contrary, L3JRC represented small fires proportionally better than large fires. Some moderate size fires were captured only by one of the algorithms, depending on the region. Overestimation in GBA2000 seemed to be more important in small central fires; however, the two VGT-derived datasets provided the best overall representation in terms of the number of fires detected.

**Figure 3:**
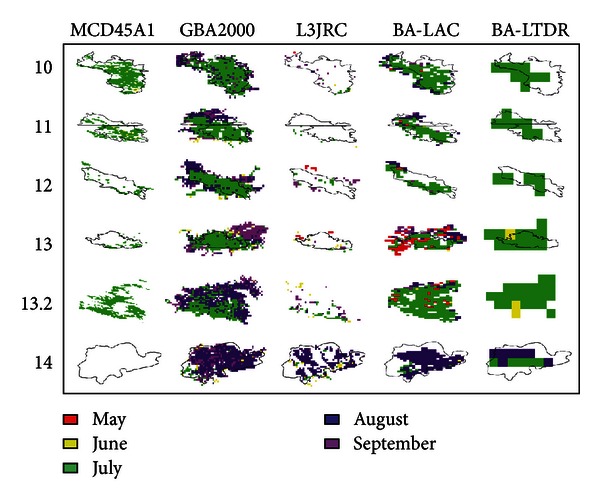
Spatiotemporal discrepancies among BA datasets (except for BA-PAL) in burn scar mapping in the mid-West region: monthly timing depiction. Pixel sizes keep the original proportion among different spatial resolutions, and color coding reflects monthly timing. Fire contours from CFSNFD polygon version are added only as means of guidance except for #13.2 where it did not exist.

Burn detections from the mid-West region in all datasets except for PAL.ba are depicted scene by scene on a monthly basis in Figure 3, where some spatiotemporal patterns may now be observed. Burn date variability within the same fire tended to be higher in VGT-derived data and LAC.ba, and it was mostly associated to burn date delay or temporal range extension. Moreover, L3JRC presented simultaneously high temporal and spatial variability, with a considerable proportion of isolated cells or groups of cells. On the contrary, MCD45A1 and LTDR.ba maintained a rather homogeneous and consistent spatiotemporal distribution for most scenes.

Some other spatiotemporal patterns were found in the rest of the regions: (a) northern fires were well represented in MCD45A1 and GBA2000 but were delayed by up to one month in the latter; (b) West coast fires—only estimated by the VGT-derived datasets—showed highly variable burn date estimates compared to other regions; and (c) heterogeneity of timing estimates in a site varied considerably in LTDR.ba between different regions (higher heterogeneity in mid-West and eastern sites).

### 5.2. Temporal Accuracy Assessment

**Figure 4:**
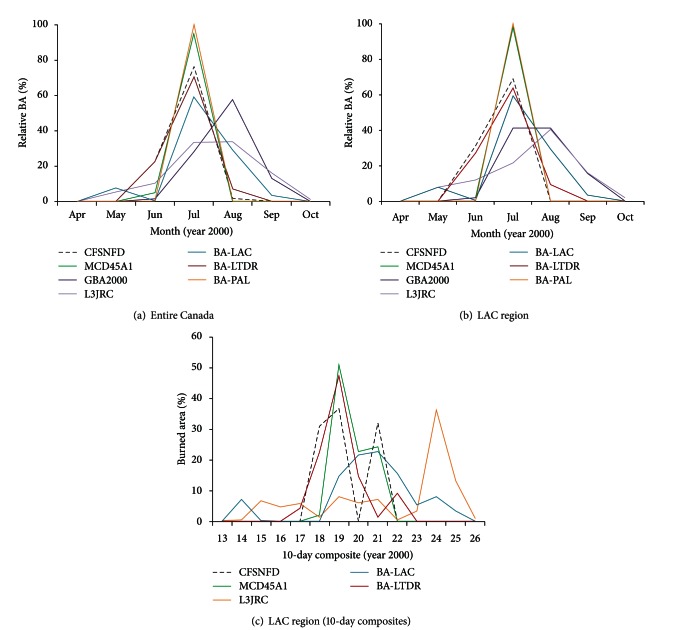
Temporal evolution of BA of Canadian fires greater than 5,000 ha during the year 2000, relative to total BA estimated by each dataset: (a) monthly, on global scale; (b) monthly, in the LAC; (c) every 10 days in the LAC for BA datasets when the temporal resolution allows it.

Monthly distributions of BA percentages were depicted in Figures 4(a) and 4(b). GBA2000 presented a monthly pattern almost matching that of the CFSNFD, except for a +1 month temporal shift. Both of the VGT-derived datasets showed a delayed maximum in August, although for the rest the maximum BA percentage was registered in July of that year. However, the most conspicuous temporal shifts were found in the LAC.ba dataset, with +1 month in 30% of the BA at the end of the fire season and −1 month in 10% during May.

With a finer temporal resolution (Figure 4(c)), BA was mainly distributed along composites 18 (June), 19, and 21 (July) according to the CFSNFD, with a major peak at the end of June and a secondary peak at the end of July. Temporal shifts range now from +1 composite—the two maxima in LTDR.ba and only the first one in MCD45A1—and up to +3 composites in LAC.ba. The time range was moderately extended in LTDR.ba and much more in LAC.ba, where nearly 40% of the area burned in the central subset was located between the 24th and 25th composites; in contrast, the MCD45A1 time range narrowed with respect to the reference data. 

## 6. Discussion 

Comparative fire-by-fire BA estimations shown in Table 3 provide an idea of accuracy in terms of BA quantification rather than actual spatial accuracy, for which validation with 30 m Landsat TM/ETM+ imagery or other moderate-resolution data might be preferable rather than the CFSNFD. Although both concepts (sensitivity and spatial accuracy) should correlate, the first one allowed us to preclude errors caused by geospatial processing, such as georeferencing issues or border effects linked to large pixels. 

### 6.1. Patch Size versus Pixel Size

According to the results shown in Tables 3 and 4, PAL.ba should never be used for BA monitoring when fires are smaller than 16,000 ha. In larger areas, underestimation ranges from −56% to −100%, and sensitivity decreases in large fires in the mid-West, indicating that there are other factors involved apart from patch size. The use of three datasets obtained from the same sensor with different pixel sizes allows us to weight the influence of the spatial resolution in BA detection. Starting with the lowest spatial resolution, the 8 km PAL.ba dataset showed an underestimation greater than −85%, in accordance with Carmona-Moreno et al. [3], who pointed out that the BA omissions for boreal forest in higher latitudes were deemed to be greater than 80%. Next, the Bayesian network algorithm used in the 5 km LTDR.ba dataset increased the total amount of BA, with an underestimation of below −60%. Three important factors contributed to these improvements are (a) improved spatial resolution, (b) improved data quality of LTDR data (calibration, geolocation, and BRDF corrections), and (c) a new algorithm used. However, the BA overall quantification does not improve much from this dataset to the 1 km LAC.ba data, in the central spatial subset, although the spatial resolution is five times more detailed in the latter.

On the other hand, MCD45A1 has the finest spatial resolution (500 m) and yet showed substantial underestimation (more than 80%) with no detections in half of the fires larger than 5,000 ha, leading us to conclude that the (fire size) : (pixel size) ratio is not necessarily the most limiting factor in BA detection. Moreover, during the same year in Southern Africa the MCD45A1 dataset only omitted 25% of the area burned [33], which is considerably better result than that in North-American boreal forest, pointing to a variable efficiency of the product in different environments.

### 6.2. Multitemporal, Multisensor, and Multispectral Approaches

Calibration problems and different operational events occurred during this year which caused the loss of a certain quantity of data available from this sensor (http://mcst.gsfc.nasa.gov/index.php?section=15) and may explain to some extent the overall BA underestimation of the MCD45A1 product. Nevertheless, several radiometric constraints are combined with temporal constraints (the temporal window of ±8 days used in change detection) and with the circumstantial lack of MODIS data that year. Pixel quality requirements might be too restrictive in this case, causing great omission of burned cells. Otherwise, the multitemporal 500 m approach (where no data compositing is needed and every valid daily data file is used in order to compute presence and persistence of sharp radiometric changes) might have been more accurate, spatially and temporally. The temporal window is longer in other datasets: GBA2000 carries out 10- to 30-day change observations, and LTDR.ba checks up to ±60-day and even pre- and postfire years observations. 

As well as the differences in the use of multitemporal data for change detection, all BA algorithms studied in this work except for MCD45A1, use spectral indices in addition to band reflectances, as they are known to reduce noise and sharpen both vegetation activity and BA signals. Most relevant indices used in LTDR.ba were GEMI, NDVI, and Vi3T as vegetation indices using the NIR and red regions, whereas the BBFI (Burned Boreal Forest Index) added the MIR region for better detection of bare soil caused by fire. However, several fires of differing sizes were not represented by any of the AVHRR-derived datasets. This probably reflects some advantage of the spectral data used in the VGT- and MODIS-derived datasets over the others. One aspect that these three products do have in common is the use of a SWIR band centered at ~1.65 *μ*m, from VGT channel 4 and MODIS channel 6, respectively. This region of the spectrum would approximately match the AVHRR channel 3A reflectance, which was not included in either of the BA algorithms working with this sensor. Moreover, the SWIR spectral regions were used in MCD45A1 span to MODIS bands 5 (1.24 *μ*m) and 7 (2.13 *μ*m), eventually increasing the probability of discrimination between different BA patterns and vegetation covers (e.g., in presence of forest discontinuities or less foliage density). In contrast, the AVHRR-derived datasets used the Red, the NIR, and—except for LAC.ba—the MWIR spectral bands; the latter is centered at 3.75 *μ*m, corresponding to the thermal AVHRR channel 3B emissivity.

On the other hand, GBA2000 somehow included both SWIR and MWIR spectral information, as it used the AVHRR active fire product (based on emissivity rather than reflectance) in addition to the VGT postfire season data. It consists of a different approach to obtain more spectral information, by means of two remote sensing sources and combining the advantages of both sensors in the detection of fire-related processes. However, it embraces pros and cons of both sources, which could be a reason why GBA2000 overestimated BA and biased the date of burn of several fires.

### 6.3. Thresholding Concept

As shown previously, PAL.ba presented the least variable total error and never detected burn scars smaller than 16,000 ha, making it the most size restricted and predictable dataset at the same time. Exceptionally, two large fires were not detected by PAL.ba (#10 and #14), which might be more related to the risk associated with the thresholding approach, rather than to its coarse spatial resolution. Fixed radiometric thresholds are usually quite tolerant in order to exclude land covers that are very different to the target feature; as such, dynamic thresholds are able to refine the selections (e.g., decrease of GEMI after fire). Although fixed and even dynamic thresholds may be adapted to the study area (e.g., North-American boreal forest), once established they may not actually adapt to the peculiarities of different sites. In several cases, undetected fires were geographically close to others that were detected, meaning that the same threshold criteria did not operate in the same way within the same area. Thus, in absence of other discriminating mechanisms (such as the Bayesian network in LTDR.ba or neighborhood tests in LTDR.ba and L3JRC), the algorithm may fail by underestimation when thresholds are too restrictive, or by overestimation in the opposite case. For example, LTDR.ba with a relatively similar spatial resolution showed more adaptability than the previous PAL.ba, by detecting seven additional regions (including sites #10 and #14) and by reducing underestimation in practically all of them. In this case, the Bayesian network could be improved by using training areas taken from data of the year analyzed, instead of using the same model for the time series as described by Moreno-Ruiz et al. [4]. However the more we increase the adaptability to a particular year, the lower its capacity to adapt to different years in the same area of study may be.

### 6.4. Geographic Variations Linked to Ecological Factors

Moving on now to analyze the ecological factors causing the geographic variability of BA detection, there seems to be a latitudinal variation of BA detection efficiency: mid-West fires are mostly detected by all the algorithms whereas North-West fires are hardly detected by a handful of them. This pattern coincides with the ecological changes from boreal forest to tundra in the transition belt between both ecosystems. Mid-West sites correspond with medium to tall, closed stands of white spruce, black spruce, and balsam fir in late successional stages. In such environment, once fire has reached the tree cover it will spread through the crowns very rapidly, releasing great energy; strong radiometric signals emitted during the fire will be easily detected in thermal bands, and the BA signal will persist longer after the fire than in an open forest, where shrub and herbaceous species are predominant and postfire recovery is faster. Therefore, BA mapping based on active fire detection may benefit from this regional feature.

The case of fire #15, undetected by all BA algorithms, is coherent with this interpretation, since the forest structure is very similar to the transition zone of northern sites: open stunted stands of black spruce with significant understory composed of ericaceous shrubs and other species such as dwarf  birch and Labrador tea. Furthermore, southern site #14, which caused detection problems in PAL.ba and MCD45A1, follows the same structural pattern, more similar to northern locations and actually placed in a different ecozone to the other mid-West sites: jack pine is the dominant tree, the understory is mostly formed by ericaceous shrubs and lichen, and earlier successional states are predominant as fires occur very often. Something similar is observed in West coast sites except for the fact that ecological particularities are caused by an altitudinal variation, as they are placed on the Cordilleran System at more than 1,500 m in height; there are open forests of white spruce, black spruce, and an understory of deciduous shrubs in addition to ericaceous, dwarf willows, and birch. Only VGT-derived datasets were able to detect them.

Finally, eastern fires were intermittently detected by different BA algorithms. The smallest eastern fire was exclusively represented in L3JRC; the largest (#18) was only reasonably well captured by GBA2000 (−17%), partially detected by PAL.ba (−56%), barely by LTDR.ba (−81%) and L3JRC (−98%), and only a trace in MCD45A1. Here, very open forests of black spruce are found, where 50% of vegetation cover is composed of nonericaceous shrubs; a transition zone to tundra and mixed evergreen and deciduous shrubs appears in the last two test sites, influenced by the Atlantic Ocean. Thus, LTDR.ba may have been more efficient in areas where the understory was not dominated by ericaceous type of shrub, whereas MCD45A1 worked in the opposite way, being more efficient in the northwestern open forests than in the eastern ones. The overall efficiency of GBA2000 can be partially caused by taking its ecozone features into account.

### 6.5. Temporal Shifts and Variability

Several factors contributed to producing temporal shifts of BA estimates with respect to the reference data temporal distribution, for instance, the unequal spatial representation of different fires. Figures 4(a) and 4(b) show how temporal shifts shorten as spatial variability lowers by subsampling (making up the central subset) allowing LAC.ba data to be added to the comparison as well. On the other hand, the fact that most fires in the central spatial subset occurred at the end of the month according to the CFSNFD may have increased the temporal shift observed at a monthly resolution (Figures 4(b) and 4(c)), especially in the case of MCD45A1 and LTDR.ba, which were the two datasets that best reproduced the reference temporal pattern. MCD45A1 was particularly influenced by the temporal resolution used, as the two major peaks fell within the same month. At site level, the spatiotemporal depiction of central fires showed that GBA2000 presented the most clearly defined and consistent shapes. However, timing differences between close pixels were up to four months, an incoherence also found in LAC.ba. In these cases, earliest and latest burn dates seemed to be linked to some peripheral distribution and spatial discrepancies with the other products. However, these two datasets agreed with the predominant date in the site core of LTDR.ba and MCD45A1, which thus showed the most consistent temporal distribution in terms of realistic burning duration of a fire and burn date variability among adjacent cells. 

## 7. Conclusions

The fire-by-fire approach in the accuracy assessment of BA datasets revealed more details about differences in BA detection than the global statistics based on a regular grid. It also avoided errors linked to georeferencing and upscaling issues. 

The ratio between fire size and pixel size is one of the factors influencing BA detection, but once over a minimum size threshold of the fire, other factors related to the BA detection method appear to be more influential. Among them are the width of the temporal window chosen for the change detection, the wavelengths used (especially in the MIR spectral region), the use of vegetation indices or BA-correlated indices, and the integration of different remote sensing sources.

Other factors are contextual and linked to particular ecosystems within the boreal forest biome, as well as to the transition zone between boreal forest and tundra. Thus, different vertical structures of the forest, species composition, fire regimes, and postfire recovery will affect the capacity for mapping the area burned. For example, highly energetic crown fires were the most common, benefiting the BA mapping methods that are based on the emissivity from active fires; however, in more open forests, less energetic surface and smoldering fires take place and different approaches are needed.

In terms of overall BA quantification, the estimated ranking of the datasets assessed in this work for the year 2000 was GBA2000 > LTDR.ba > MCD45A1 > L3JRC > PAL.ba. The first one also showed, however, the highest error variability and occasional overestimation, whereas the other VGT-derived dataset L3JRC, in spite of its rather poor BA estimation in the study area, was able to capture areas that passed unnoticed to others. Thus, regarding the overall representativeness of BA sites in the entire territory, our ranking would change with L3JRC in the first place. The MODIS-derived dataset was not the best BA estimator in year 2000, mostly caused by a circumstantial lack of data, but possibly also due to a lower adaptability of its change detection method to the boreal forests of Canada compared to other datasets. Regarding the AVHRR-derived datasets, BA estimations generally improved from coarser to finer spatial resolution, particularly from 8 km to 5 km; however, there was no substantial improvement from 5 km to 1 km, indicating that differences in the classification method between LTDR.ba and LAC.ba were more important than pixel size.

On the other hand, from a temporal accuracy perspective, burn date results in increasing order of spatiotemporal coherence and temporal accuracy was MCD45A1 > LTDR.ba~PAL.ba > LAC.ba > GBA2000 > L3JRC. The LTDR.ba dataset struck a balance that year between MCD45A1 (spatial and temporal accuracy but great omission) and GBA2000 (best BA quantification but local overestimation and burn date retardation). For the rest of the datasets assessed, the higher the spatial accuracy is, the lower the temporal accuracy, and *vice versa*.

## Figures and Tables

**Table 1:** Original datasets features grouped by sensor. Spectral bands in bold are involved in the corresponding burn scar detection algorithms.

Dataset	Spatial		Temporal		Spectral		
	Coverage	Resolution	Coverage (BA time series)	Resolution (Date of burn)	Spectral bands used (2000)*	Compositing criteria	BA detection
**AVHRR**							
BA-PAL	Global	0.1° (~8 km)	1984–2000	Monthly	***ρ*1 (~0.63 *μ*m)**	7-day composites; minimum albedo	Multitemporal and multithreshold BA Algorithm (BAA) [2]
					***ρ*2 (~0.83 *μ*m)**		
					**T3 (~3.75 *μ*m)**		
					T4 (~10.8 *μ*m)		
					T5 (~12.0 *μ*m)		
BA-LTDR	Global	0.05° (~5 km)	1984–2000	10 days	***ρ*1 (~0.63 *μ*m)**	10-day composites; maximum T3	Bayesian network classification [4]**
					***ρ*2 (~0.83 *μ*m)**		
					*ρ*3 (~1.61 *μ*m)		
					**T3 (~3.75 *μ*m)**		
					T4 (~10.8 *μ*m)		
					T5 (~12.0 *μ*m)		
BA-LAC	Canada, northern USA, Alaska, and Greenland	0.01° (~1 km)	1984–2006	Daily	***ρ*1 (~0.63 *μ*m)**	10-day composites; SPARC algorithm [6]	Multitemporal and multithreshold contextual algorithm [5]**
					***ρ*2 (~0.83 *μ*m)**		
					T3 (~3.75 *μ*m)		
					T4 (~10.8 *μ*m)		
					T5 (~12.0 *μ*m)		
**VEGETATION**							
GBA2000	Global	0.01° (~1 km)	2000	Monthly	B1 (~0.45 *μ*m)	10-day composites; maximum NDVI	Multiple logistic regression model [7]**
					**B2 (~0.66 *μ*m)**		
					**B3 (~0.83 *μ*m)**		
					**B4 (~1.65 *μ*m)**		
L3JRC	Global	0.01° (~1 km)	2000–2007	Daily	B1 (~0.45 *μ*m)	None	Comparison of daily values and intermediate composites in NIR [8]
					**B3 (~0.83 *μ*m)**		
					B4 (~1.65 *μ*m)		
**MODIS**							
MCD45A1	Global	(~500 m)	2000–present	Daily	1 (~0.65 *μ*m)	None	Bidirectional Reflectance Distribution Function (BRDF) [9]
					**2 (~0.86 *μ*m)**		
					3 (~0.47 *μ*m)		
					4 (~0.56 *μ*m)		
					**5 (~1.24 *μ*m)**		
					**6 (~1.64 *μ*m)**		
					**7 (~2.13 *μ*m)**		

*Bands in bold were used in change detection.

**BA detection was optimized for the Canadian boreal region.

**Table 2:** Mean and standard deviation of relative errors in BA estimations in all BA estimates for the year 2000, for Canada and the LAC region (fires larger than 5,000 ha).

	Entire Canada		Local Coverage subset	
	*µ*	*σ*	*µ*	*σ*
MCD45A1	−82.66	21.51	−75.73	22.4
GBA2000	−16.3	41.57	3.92	38.49
L3JRC	−79.41	20.43	−78.47	18.00
BA-LAC	—	—	−34.04	28.14
BA-LTDR	−58.94	34.2	−35.48	28.54
BA-PAL	−86.89	17.67	−85.6	15.41

**Table 3:** Comparative BA estimates in fires larger than 5,000 ha that occurred in the year 2000, for all datasets in a Lon/Lat WGS84 reference system.

Scene ID	BA estimations (ha)						
	CFSNFD	MCD45A1	GBA2000	L3JRC	BA-LAC	BA-LTDR	BA-PAL
LAC region							
2	5 450	0	1 903	1,485	0	0	0
8	6 500	0	4 523	4,744	0	0	0
9	7 693	0	0	802	0	0	0
10	20 040	11 116	24 761	1,391	16 928	13 107	0
11	16 452	6 432	22 340	496	11 353	10 904	6 000
12	9 249	1 737	18 394	2,225	6 651	7 765	0
13	19 154	1 436	24 112	1,494	18 764	18 776	6 026
13.2	43 701	20 081	45 023	6,044	36 391	37 943	12 181
14	39 850	0	33 628	17,502	20 790	19 963	0

Subtotal	168 089	40 802	174 684	36,184	110 877	108 458	24 207

1	5 668	0	5 751	0		0	0
1.1	9 000	0	2 501	266		0	0
2.1	6 800	636	0	2,287		0	0
2.2	5 800	272	0	0		0	0
3	6 500	1 762	0	2,342		2 578	0
4	15 600	2 805	10 633	6,973		0	0
5	7 500	3 588	5 959	839		0	0
6	8 000	1 044	6 381	4,930		0	0
7	15 500	8 898	15 590	8,155		5 333	0
15	5 182	0	0	0		0	0
16	13 288	0	13 598	59		9 194	0
17	7 483	0	0	298		0	0
18	48 079	70	39 977	872		9 118	21 070
19	10 125	0	5 197	1,285		0	0
20	12 636	0	8 704	6,612		7 093	0

Total	339 582	59 877	288 975	71,102	110 877	141 774	45 277

**Table 4:** Proportion of BA estimates with respect to BA registered in CFSNFD, presenting different detectability patterns among BA products related to the location of fires within the Canadian territory.

	Regional BA estimates with respect to CFSNFD (%)				
	West coast (WC)	North-West (NW)	Mid-West (mW)	Central Canada (cC)	East coast (EC)
MCD45A1	0	24	26	0	<1
GBA2000	56	58	108*	0	74
L3JRC	1	20	41	0	10
BA-LAC	—	—	71	—	—
BA-LTDR	0	10	69	0	28
BA-PAL	0	0	16	0	23

*GBA2000 overestimation of total BA in mW.
